# Comorbidity associated with worse outcomes in a population of limited cochlear implant performers

**DOI:** 10.1002/lio2.985

**Published:** 2022-12-16

**Authors:** Erika Lee, Justyn Pisa, Jordan Hochman

**Affiliations:** ^1^ University of Manitoba Faculty of Medicine Otolaryngology ‐ Head and Neck Surgery Winnipeg Manitoba Canada; ^2^ University of Manitoba Faculty of Medicine Surgical Hearing Implant Program, Department of Otolaryngology‐Head and Neck Surgery Winnipeg Manitoba Canada; ^3^ University of Manitoba Faculty of Medicine Neurotologic Surgery, Department of Otolaryngology ‐ Head and Neck Surgery Winnipeg Manitoba Canada

**Keywords:** audiology, cochlear implants, sensorineural hearing loss

## Abstract

**Introduction:**

Most patients significantly benefit from cochlear implantation (CI). However, speech understanding varies widely, with a small proportion of patients demonstrating limited audiometric outcomes. While there are well‐documented determinants of poor performance, there remains a cohort of patients that do not meet expected outcomes. Preoperative prognostication is desirable to manage expectations, ensure value of the intervention, and reduce risk. The objective of the study is to evaluate variables found within a single CI center's most limited functioning cohort following implantation.

**Methods:**

A retrospective review of a single CI program's cohort of (344 ears) patients implanted between 2011 and 2018 whose 1‐year postimplantation AzBio scores fall 2 SDs below the mean was performed. Exclusion criteria includes skullbase pathology, pre/peri‐lingual deafness, cochlear anatomic abnormalities, English as an additional language, and limited electrode insertion depth. Overall, 26 patients were identified.

**Results:**

The study population's postimplantation net benefit AzBio score is 18% compared to the entire program's 47% (*p* < 0.05). This group is older (71.8 vs. 59.0 years, *p* < 0.05) with a longer duration of hearing loss (26.4 vs. 18.0 years, *p* < 0.05) and with a lower preoperative AzBio score [14% lower (*p* < 0.05)]. A host of medical conditions were identified in the subpopulation, with a trend towards significance in those suffering from either malignancy or cardiac condition. Escalating comorbid status was associated with worse performance (*p* < 0.05).

**Conclusion:**

Within a cohort of limited‐performing CI users, benefit tended to decrease with escalating number of comorbid conditions. This information may serve to inform preoperative patient counseling.

**Level of evidence:**

Level IV (evidence from a case control study).

## Graphical abstract

Many patients receive benefit from cochlear implant surgery, however a cohort of patients remain that do not perform as expected with age and duration of hearing loss the primary risk factors.

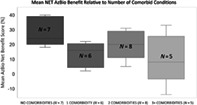

## 1 INTRODUCTION

Cochlear implantation (CI) is a common treatment for severe‐to‐profound hearing loss that involves direct electrical stimulation of the auditory nerve. It is quite successful in partial hearing restoration. However, differences in functional outcomes are common.^1^, ^2^, ^3^ Speech understanding varies and a small proportion of recipients perform poorly on formal outcome measures.^4^, ^5^ While there are well‐documented determinants of poor performance, a better understanding of these factors can facilitate preoperative prognostication of outcome and patient counseling.

A number of variables are well appreciated to be associated with more limited functional outcomes. Age at implantation and duration of hearing loss are correlated with lower postoperative hearing scores.^1^, ^2^, ^3^, ^4^, ^5^, ^6^, ^7^, ^8^, ^9^, ^10^, ^11^, ^12^, ^13^, ^14^, ^15^ However, while an association with age is illustrated, there are mixed outcomes and causation may be multifaceted.^6^, ^10^, ^16^, ^17^ The issue is complex as age is associated with changes in cognition, reductions in neural plasticity, limited social activity, and comorbid status.^18^, ^19^ A similarly complicated arrangement is seen with disparate etiologies of hearing loss, cochlear nerve diameter, and electrode insertion dynamics.^1^, ^2^, ^3^, ^4^, ^5^, ^6^, ^7^, ^8^, ^9^, ^10^, ^11^, ^12^, ^13^, ^14^, ^15^, ^16^, ^17^, ^18^

In general, the incidence of medical issues increases with age. The effect of comorbidities on CI outcomes has not been well delineated. One study found that older patients undergoing CI tended to have more comorbidities, however, did not differentiate audiometric outcomes based on health.^18^ Outcomes specifically comparing comorbid burden to audiometric outcomes and quality‐of‐life (QOL) measures were not addressed. Furthermore, the import of disparate types of medical conditions on audiometric outcomes is not clear.

QOL has been shown to improve in both younger and older CI patients, although studies used varied QOL instruments.^20^, ^21^, ^22^, ^23^, ^24^ Progressive hearing loss in adults is associated with reduced QOL due to communication barriers and decreased speech recognition.^25^, ^26^ There is some evidence that speech understanding increases postoperatively and patients report significant improvement, however, outcomes were influenced by factors, such as educational level, co‐existing depression, and preoperative working memory.^20^, ^21^, ^23^

The objective of the study is to determine and evaluate variables that may assist in forecasting outcomes and communicating expectations in patients that are anticipated to have challenged postoperative speech discrimination following CI (older age, longer duration of deafness, and modest preoperative function).

## 2 METHODS

A retrospective analysis of a group of adult patients who gained the least benefit after CI in a single program's cohort of patients implanted between 2011 to 2018 was performed. A total of 344 ears were implanted during this time period. Inclusion criteria included those whose postimplantation AzBio speech perception scores at 1‐year fell two standard deviations below the mean. Exclusion criteria included any skullbase pathology, pre/peri‐lingual deafness, cochlear anatomic abnormalities, English as an additional language prohibiting accurate speech scores, and limited electrode insertion depth. Approval was received from the Research Ethics Board at University of Manitoba prior to any study activity (HS18623 (H2015:209)).

Peri‐operative data collection included patient questionnaires, imaging characteristics, and surgical findings. Patient self‐account of health status and review of an inclusive provincial electronic record were collected. Postoperative speech recognition scores are measured annually, employing the AzBio speech perception test. Outcomes were compared to the general cohort of 344 patients. Statistical analysis was conducted using two‐tailed T‐test.

Secondary outcomes include quality of life questionnaires from the International Outcome Inventory for Cochlear Implants (IOI‐CI) routinely collected from patients. Quality of life measures were analyzed using Mann–Whitney Test.

## 3 RESULTS

A total of 31 patients were identified and 26 patients were ultimately included in analysis. One patient with Neurofibromatosis type 2 and four prelingually deafened patients were removed from the study. There were four patients deceased at time of analysis.

The study cohort's average preoperative AzBio score is 12%, the postoperative scores at 1 year are 30%, with a net benefit of 18%. In the general cohort, the preoperative AzBio score is 27%, the postoperative score at 1 year is 74% and net benefit is 47% (Table 1). Preoperative AzBio scores are 14% lower (*p* < 0.05) than the general population. The postimplantation net benefit AzBio score at one year was also significantly different (47% compared to 18%, *p* < 0.05).

**TABLE 1:** Patient word scores found in the general cohort and study subpopulation

	Study subgroup (*n* = 26)	General cohort (*n* = 344)	*p*‐value
Pre‐Op AzBio score	12% (±14%)	27% (±20%)	<0.05
Post‐Op AzBio score	30% (±12%)	74% (±17%)	<0.05
Net Benefit AzBio score	18% (±12%)	47% (±18%)	<0.05
Age at implantation (years)	71.2 (±12.7)	59.0 (±15.9)	<0.05
Duration of hearing loss (years)	26.4 (±13.9)	18.7 (±16.5)	<0.05

The average age at implantation of the study subgroup was significantly older (71.2 vs. 59.0 years, *p* < 0.05) than the general CI population (Table 1). Duration of hearing loss in the study subgroup was significantly longer (26.4 vs. 18.0 years, *p* < 0.05). The ratio of male: female in the study subgroup was 10:16. Males had a mean post‐op AzBio score of 25% and net benefit of 11% compared to females, with a mean AzBio score of 31% and net benefit of 21%. There were no significant differences.

The presence of comorbid conditions was reviewed. The type of condition did not significantly influence postoperative function (Table 2). A nonsignificant difference is identified in the net benefit AzBio scores in individuals with either cancer or cardiac condition (Table 2).

**TABLE 2:** A comparison of AzBio scores by comorbid condition

	Pre‐Op AzBio score	Post‐Op AzBio score	Net benefit AzBio score
Study subgroup (*n* = 26)	12% (±14%)	30% (±12%)	18% (±12%)
No comorbidities (*n* = 7)	5% (±7%)	32% (±13%)	27% (±9%)
Cardiac condition (*n* = 9)	15% (±18%)	25% (±13%)	10% (±14%)
Otologic condition (*n* = 7)	15% (±17%)	28% (±16%)	13% (±10%)
Neurologic condition (*n* = 6)	11% (±21%)	25% (±13%)	14% (±17%)
Autoimmune condition (*n* = 9)	10% (±12%)	31% (±8%)	20% (±8%)
Cancer (*n* = 4)	27% (± 23%)	38% (± 6%)	11% (± 20%)
Renal condition (*n* = 3)	0%	21% (±18%)	21% (±18%)

**FIGURE 1:**
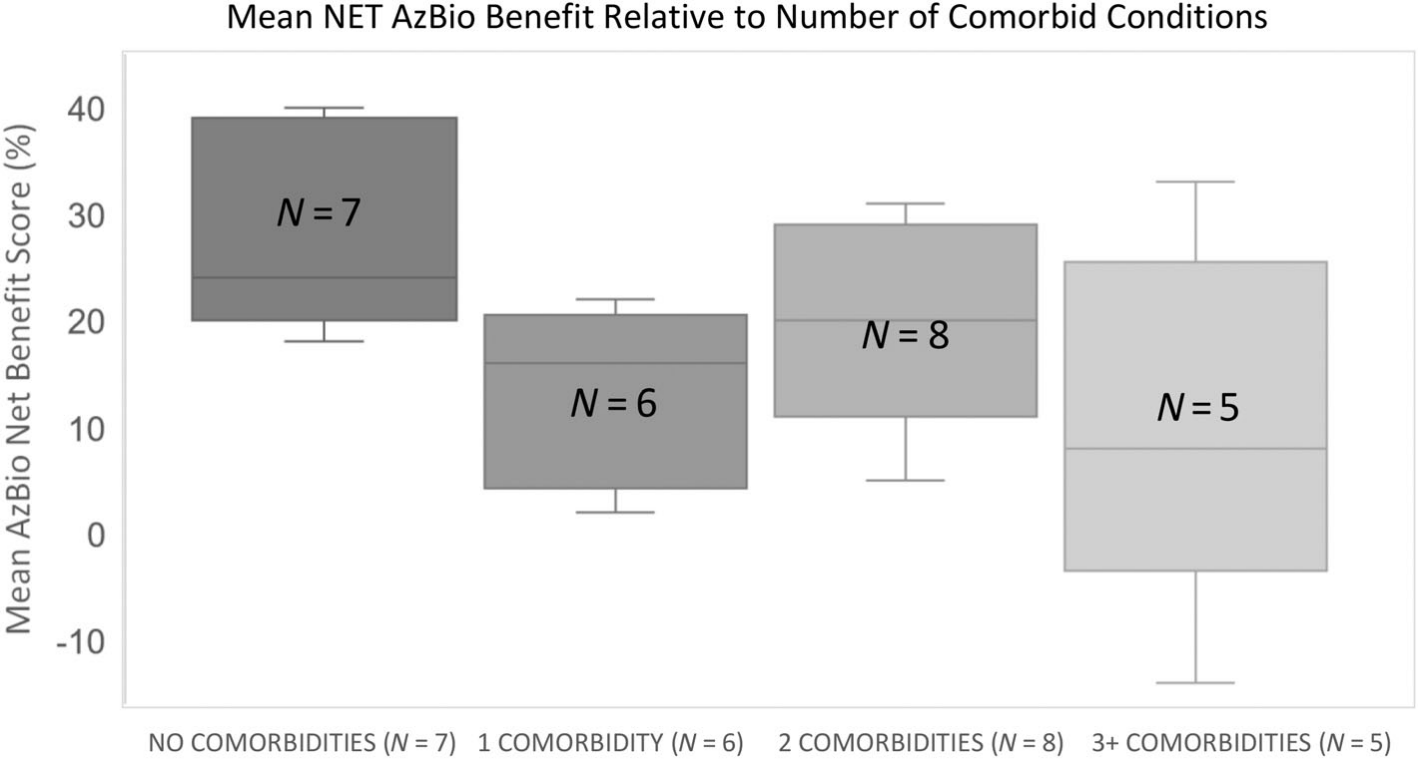
Contrasting the number of comorbid conditions to postoperative Net AzBio score

An inverse relationship is illustrated when contrasting audiometric outcomes and number of comorbid conditions (Figure 1). Differences were found between nonmorbid and those with one comorbidity, and those with three or more comorbidities (*p* < 0.05). Having two conditions illustrated a trend to worse function without statistical significance.

**FIGURE 2:**
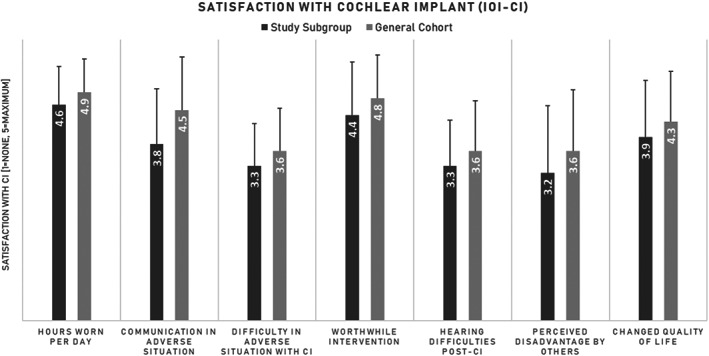
Population satisfaction with cochlear implantation compared to the entire cohort

The average responses to the QOL survey from the study cohort did not differ from the general cohorts (Figure 2).

## 4 DISCUSSION

This study investigated factors associated with limited outcomes in a cohort of patients who performed 2 SDs below the average postoperative AzBio speech perception score. The determination to examine only those 2 SDs from the mean performance was arbitrary, but with the intent to address those with the most limited functional outcomes.

### 4.1 Comorbidity

The occurrence of multimorbidity increases with age and prevalence ranges from 55% to 98% in persons 60 years and older.^19^ The majority of the study cohort, 19 out of 26 participants, had one or more comorbidities. This study found that the greater the number of comorbid conditions tended to correlate with more limited performance. This was independent of age. This is not unanticipated. Neurologic function, and ischemia are both a correlate for cogitation. With increasing comorbidities come polypharmacy, heightened psychological stress, and decreased quality of life, all of which are factors found to have association with lower implant audiological measures.^18^, ^25^, ^27^ However, there is also developing literature illustrating that hearing rehabilitation has a very positive impact on validated cognitive instruments.^28^, ^29^, ^30^

Type of comorbid conditions (cardiac, otologic, neurologic, rheumatologic, renal, and cancer) was not a significant determinant of function; however, patients with cardiac conditions and cancer trended to worse outcomes.

An explanation of outcomes of patients with cardiac conditions is likely owing to vascular health and the determinants and associations with vascular health, with central demyelination, neuronal density, and recruitment following implantation.^31^ The discrepancy found in patients with historic cancer could relate to iatrogenic inner ear damage (skullbase radiation or ototoxic exposures).^32^, ^33^ However, all treatment modalities for cancer impact central function with possible deleterious effects on postimplantation speech intelligibility.

It was not possible to correct for age in reviewing the implications of associated health conditions in this subpopulation as the cohort size was relatively small. Furthermore, a significant caveat is that it was not possible to differentiate the degree or significance of any given illness. As an illustration, the import of paroxysms of SVT on a patients' microangiopathic health is likely different from having previously undergone a five‐vessel bypass graft following years of ischemia.

### 4.2 Age, duration of hearing loss and preoperative function

Similar to other work, this study found all three variables to be associated with worse overall function. The study population experienced a longer duration of hearing loss, a finding that is echoed in previous studies.^1^, ^2^, ^6^, ^7^, ^8^, ^9^, ^10^, ^11^, ^12^, ^13^, ^14^ The cohort is also significantly older than the general cohort. It is assumed that the degenerative process of aging (multifaceted impact) and lengthier auditory deprivation leads to a negative correlation with postoperative hearing outcomes. This is perhaps through auditory nerve fiber atrophy, damage to hair cells and stria vascularis, or reduced plasticity of the auditory pathways or auditory cortex.^26^, ^34^, ^35^

In addition, the definition of duration of deafness is not consistent across reports, ranging from age at implantation subtracted from reported onset of deafness, to reported years of hearing loss.^1^, ^2^, ^6^, ^7^, ^8^, ^9^, ^10^, ^11^, ^12^, ^13^, ^14^ In this study, duration of hearing loss is measured via patient‐reported years.

The subgroup entered into surgery with less hearing as evidenced by lower preoperative AzBio scores compared to the general population. This variable is likely highly associated with age at implantation and duration of deafness. Within the confines of this study, it was not possible to analyze as an isolated variable.

### 4.3 Quality of Life

QOL did not significantly differ between the general CI cohort and the study population. In general, the study population with unexpected outcomes tended to have similarly improved QOL as those with normative performance. This is arguably equally important for patients to audiometric outcomes. A recent study found that patients reported improved QOL following CI which did not necessarily correlate with popular clinical measures of speech recognition.^24^

This feature is humbling and reminds implant centers of the need to be patient‐centric in decision making.

There are several study limitations. The small sample size may have profound implications when generalizing the impact of comorbid conditions on function. Furthermore, the cohort size did not have power to allow for multivariate analysis, nor adjustments for age at implantation or duration of deafness. Other confounding factors (i.e., hearing aid fit, neurocognition, psychological factors) would likely have influence, however, sufficient data was not available. Future work should attempt to obtain more granular detail regarding individual conditions. It would be interesting to assess for medical conditions in the broader CI database for a similar finding of more limited objective function with the increasing number of comorbid conditions.

## 5 CONCLUSION

Within a population of limited performing CI users, objective benefit declined with more comorbid health. This information should inform preoperative patient counseling.

## CONFLICT OF INTEREST

The authors declare that they have no competing interests.
